# Effect of gamma rays on the essential oil and biochemical characteristics of the *Satureja mutica* Fisch & C. A. Mey

**DOI:** 10.1038/s41598-024-57989-w

**Published:** 2024-03-30

**Authors:** Fatemeh Mahdi Navehsi, Vahid Abdossi, Bohloul Abbaszadeh, Razieh Azimi, Marjan Dianat

**Affiliations:** 1 Department of Horticultural Science, Science and Research Branch, Islamic Azad University, Tehran, Iran; 2 Research Institute of Forests and Rangelands, Agricultural Research Education and Extension Organization (AREEO), Tehran, Iran; 3 Department of Agronomy, Science and Research Branch, Islamic Azad University, Tehran, Iran

**Keywords:** *Satureja mutica*, Carvacrol, Essential oil, *p*-Cymene, Biological techniques, Plant sciences

## Abstract

There are 16 species in the genus *Satureja* L. (Lamiaceae), of which 10 are native. This research aimed to investigate the effect of gamma rays and storage conditions and duration on the percentage and components of the essential oil and some biochemical characteristics of *Satureja mutica* Fisch & C.A. Mey at the Research Institute of Forests and Rangelands. Plants were collected at the full flowering stage and exposed to different doses of gamma rays (0, 2.5, 5, 7.5, and 10 kGy) at the Atomic Energy Organization, Iran. The samples were kept in a refrigerator (4 °C) and in the shade (25 ± 2 °C) for 0, 120, and 240 h. This experiment was performed in a completely randomized design. Essential oil extraction was done by water distillation for 2 h. The composition of their essential oil components was identified using GC and GC/MS. Some biochemical traits, including phenol content, antioxidant capacity, and carbohydrate content, were measured. The results indicated that irradiation on the percentage of essential oil showed a statistically significant difference. In addition, the interaction effect of irradiation × storage conditions, irradiation × duration of storage, on the percentage of essential oil was significant. According to a comparison of the means, 2.5 kGy irradiation produced the highest percentage of essential oil (0.4%); in contrast, a significant decrease was detected in components with 7.5 and 10 kGy irradiation. It was observed that the percentage of some essential oil compounds decreased with the gamma-ray intensity increase. 2.5 kGy of gamma rays and shade storage conditions for 240 h led to the highest content of *p*-cymene and carvacrol. Nevertheless, the highest thymol content was obtained under refrigeration conditions without irradiation. The maximum phenol content and antioxidant capacity were obtained when the plants were irradiated with 2.5 and 7.5 kGy gamma rays. However, the maximum carbohydrate rate was observed in non-irradiated plants. It was concluded that low-intensity gamma rays could improve the percentage of essential oil and main components like *p*-cymene and carvacrol in *S. mutica* Fisch & C.A. Mey.

## Introduction

*Satureja mutica* (savory) is one of Iran's native medicinal plants, distributed in the northern regions from North Khorasan to Gilan^1^. They are perennial shrubs that bloom from summer to fall and reach a height of 30 to 50 cm. Generally, the aerial parts of the plant, which are usually harvested at the flowering stage, are rich in essential oil and used in the food, drink, and perfume industries^2^. *S. mutica* contains diverse biologically active constituents such as essential oils, tannins, triterpenes, and flavonoids. The main phenolic compounds in its essential oil are thymol and carvacrol, which are of interest in the pharmaceutical, health, and cosmetic industries^3^. Many savory species are multipurpose, having aromatic and medicinal properties. Different types of savory are widely recognized for their antioxidant, pain-relieving, anti-infectious, anti-fungal, and anti-viral activities. In traditional medicine, it is used to treat chronic diseases, such as colic, muscle pain, nausea, indigestion, diarrhea, and infectious diseases^4^.

As one of the main sources of drugs, medicinal plants have economic importance. Historically, the market could be supplied with enough medicinal plants by collecting them from nature^5^. Essential oils have multiple health benefits, primarily due to their antimicrobial and anti-inflammatory effects. Cinnamon oil's mechanism of action alters bacterial membranes, modifies lipid profiles, and inhibits cell division, protecting against colitis. On the other hand, a significant improvement was observed in the diastolic pressure of patients with metabolic syndrome when supplementing them with cumin essential oil ^6^. So, EOs contribute to disease prevention through different mechanisms of action. It has been shown that the anti-inflammatory components of EOs inhibit free radicals that cause DNA mutations. Likewise, when there is prolonged oxidative stress, an excessive accumulation of reactive oxygen species (ROS) can trigger chronic disorders such as metabolic syndrome, cardiovascular diseases, diabetes, and even cancer for the same reason that DNA mutations occur. EOs rich in polyphenols and their antioxidant properties act as therapeutic agents for these diseases ^7^.

During the past years, several widely used species have been threatened with extinction^8^. The increase in population and need for food and drugs make modern agriculture even more evident. This sudden rise in demand is due to awareness of modern drugs' harmful effects, leading to the over-exploitation of wild plants to an extent where many are endangered, threatened, vulnerable, and on the brink of extinction. So, it is imperative not to waste plant material and preserve the quality of the harvested crop ^9^. Medicinal plant quality depends on geographical origin, species, variety, and growth stage at the time of collection and transportation after harvesting, drying method, and storage method ^10^. Drying is a key element that prepares herbs and spices for u**se**. Post-harvest processing of medicinal plants is critical to preserving their therapeutic qualities, ensuring product safety, and promoting sustainability. It involves drying, cleaning, and storage procedures to maintain plant integrity and prevent contamination ^11^.

In 1969, the Food and Agriculture Organization (FAO) of the United Nations and the International Atomic Energy Agency (IAEA) reached an agreement concerning its use in agriculture and marked a turning point in agriculture development through nuclear technology. Radiation intensity is typically divided into three categories: low (less than 3 kGy), medium (between 3 and 10 kGy), and high (above 10 kGy^12^. In this regard, Ibrahim et al. ^13^ on the effect of gamma radiation on sesquiterpene activity in *Thapsia. garganica* demonstrated that neither the phytochemical profile of plant extracts nor their antioxidant activities are impacted by gamma radiation. Fennel growth characteristics and productivity were also examined in another study using gamma radiation. The findings indicated that seed irradiation increased the growth period, decreased the germination percentage, and decreased the number of leaves, the number of roots, the fresh and dry weight of the plant, and the weight of 1000 seeds ^14^. In terms of microbial restriction, phenol content, and radical scavenging activity, the effects of gamma radiation on the extract and dried leaves of three medicinal plants were examined, and the findings were inconsistent. It was found that doses of 6–12 kGy and 9–13 kGy for dry plants effectively enhanced the quality of *Euodio malayana extract.* In addition, gallic acid concentration increased in treated leaves. Gamma treatment before planting is one of the most efficient ways to increase German chamomile output, essential oil components, and chemical compounds ^15^.

Like other medicinal plants, savory's post-harvest process is critical to the production cycle. The main purpose of the drying process is to reduce the water level to less than 15% to inhibit microbial growth and to minimize biochemical changes such as flavor volatiles, color, and structural integrity of the herb ^16^. It is possible to increase plant yields per unit area by utilizing contemporary scientific approaches, such as nuclear technology and irradiation, which can increase plant yield with minimal energy consumption. Although the effects will vary depending on the product and its application, using gamma rays to apply physiological and biochemical changes, including changes in the amount of essential oil, the effect on enhancing plant production appears to be a novel solution ^17^. Literature searches indicated that the oils and biochemical of *S. mutica* Fisch. & C. A. Mey. have not been the subject of previous studies. Therefore, the present work was designed to assess the effects of γ-radiation, conditions, and duration of storage on the extraction percentage and chemical composition of essential oils isolated from *S. mutica* Fisch & C.A. Mey.

## Material and methods

### Plant materials and treatments

Plant samples were collected from the Institute of Forests and Rangelands of the country's research farm (Alborz Providence), which is situated in Karaj at 35.8439° N, 50.9715° E. The area receives about 235 mm of rainfall, and minimum and maximum temperatures are −20 °C and 38 °C, respectively ^18^. The specimen was presented in the central herbarium of Tehran University (Herbarium Code: TUH) and authenticated by botanist Dr. Moradi ^19^. Samples were transferred to the Atomic Energy Organization to be treated with gamma rays. After being exposed to gamma radiation at rates of 0, 2.5, 5, 7.5, and 10 kGy ^15^, the samples were kept in plastic bags in the refrigerator at 4 °C and room temperature at 25 °C for 0, 120 and 240 h. After the anticipated storage period has passed, the essential oil must be extracted, and the relevant samples must be frozen to test the biochemical features.

### Essential oil extraction and GC and GC/MS analysis

The essential oil was extracted by water distillation with Clevenger in 3 repetitions for 2 h. The essential oils were dehydrated in sodium sulfate, and after weighing the essential oil samples, the essential oil yield was reported as w/w. Next, essential oils were analyzed using GC and GC/MS devices in the plant chemistry laboratory of the Research Institute of Forests and Rangelands ^19^. An ultra-fast gas chromatograph (GC-FID) from the Thermo-UFM brand was used, equipped with a FID detector and data processor with Chrom-card 2006 software. The column was semi-polar DB-5 (5% diphenyl and 95% dimethylpolysiloxane), with a length of 10 m, an inner diameter of 0.1 mm, and a stationary phase layer thickness of 0.4 µ. The injection chamber temperature was set at 280 °C, and the detector temperature was set at 280 °C. The thermal programming of the column included raising the temperature from 60 to 285 °C with an increase rate of 40 °C/min and then keeping it at 285 °C for 3 min. Helium was used as a carrier gas at 0.5 mL/min. An Agilent 7890A gas chromatograph connected to an Agilent 5975C quadrupole mass spectrometer (USA), equipped with DB-5 column (length 30 m, inner diameter 0.25 mm, and stationary phase layer thickness equal to 0.25 µ) for qualitative analysis was used. The thermal programming of the column includes increasing the temperature from 60 to 220 °C with an increased rate of 3 °C/min and then increasing it to 260 °C with an increased rate of 20 °C/min, and finally, it is kept at this temperature for 3 min. The injection chamber was set at 260 °C and the transfer line at 280 °C. Helium was used as a carrier gas with a flow rate of 30.6 cm/s. The scanning time was one second, and the ionization energy was 70 electron volts and 40 to 340 *m/z*. The inhibition period, Quats inhibition index, mass spectra comparison with standard compounds, and employing device library information were used to identify constituents in essential oils ^20^.

#### Biochemical parameters determination

Furthermore, several biochemical parameters were measured, including antioxidant activity, phenol, and carbohydrate content.

#### Phenol content

Folin-Ciocalteu's phenol reagent was used to measure total phenol content. The extract was prepared at a concentration of 10 ml/mg, and then 0.5 ml of each extract was mixed with 2.5 ml of 0.2 normal Folin-Ciocalteu's phenol reagent and stirred for 5 min. Then, a 2 ml sodium carbonate solution of 10% at 75 g/l was added. The absorbance of the samples was measured after leaving them at room temperature for 2 h with an ultraviolet spectrophotometer at a wavelength of 760 nm against a blank (methanol). The amounts of total phenol in the extract are measured using a standard curve based on mg gallic acid /g of extract ^21^.

#### Carbohydrate content

Carbohydrates were extracted using the 95% ethanol and sulfuric acid method. For this purpose, 0.2 g fresh leaf tissue was heated with 10 cc of 95% ethanol for one hour in a Bain-Marie bath at 80 °C. One cc of 0.5% phenol and 5 cc of 98% sulfuric acid were added to each cc of this sample. The amount of absorbed light was measured at 483 nm using a spectrophotometer. The amount of extracted carbohydrates was obtained based on micrograms of glucose per gram of weight from the standard table.

#### DPPH free radical inhibition

Evaluation of leaf extract antioxidant activity was done by using DPPH method. For this purpose, first, 2.5 ml of methanolic solution of leaf extract was poured into a test tube. Then, 1 ml of methanolic DPPH solution was added to it. The contents of each tube were thoroughly vortexed. After 30 min, at room temperature and in a dark environment, their absorbance at 518 nm was read with a spectrophotometer against a blank containing methanol. In this method, vitamin C and BHT were used as positive controls. DPPH free radical inhibition percentage was calculated using the following equation:

$${\text{I}}\left( \% \right) = { 1}00 \times \, \left( {{\text{A}}_{0} - {\text{A}}_{{\text{s}}} } \right)/{\text{A}}_{0}$$

where A0 is the absorbance of the control and As, is the absorbance of the sample. The results were expressed as IC50 (an amount of antioxidant required for DPPH concentration to reach50% of the initial value) ^22^.

### Statistical analysis

The sources of variation include gamma radiation intensity at 4 levels: 0, 2.5, 7.5, and 10 kGy, storage conditions at 2 levels: room temperature of 25 °C and refrigerator at 4 °C; and storage duration factor at 3 levels: 0, 120 and 240 h. As a result, the number of studied treatments, including eight repetitions for each treatment, was equal to 92 experimental units, which were analyzed in a factorial format using a completely random design. The data in this study are presented as mean values + standard deviation (S.D.) based on three replications, analyzed, and compared using the LSD test at 0.05.

### Ethical statement

This research received no specific grant from any funding agency in the public, commercial, or not-for-profit sectors. The authors declare that there is no conflict of interest regarding this article. All authors contributed to the study's conception and design. Material preparation, data collection, and analysis were performed by [Fatemeh Mahdi Navehsi], [Vahid Abdossi], [Bohloul Abbaszadeh], [Razieh Azimi] and [Marjan Dianat]. The first draft of the manuscript was written by [Vahid Abdossi], and all authors commented on previous manuscript versions. All authors read and approved the final manuscript. Vahid Abdossi and Bohloul Abbaszadeh: Conceptualization, Methodology, Formal analysis and investigation; Vahid Abdossi: Writing-original draft preparation; Fatemeh Mahdi Navehsi, Vahid Abdossi, Bohloul Abbaszadeh**,** Razieh Azimi and Marjan Dianat: Writing-review and editing; Vahid Abdossi and Bohloul Abbaszadeh: Resources and supervision. All methods were conducted following the relevant guidelines/regulations/legislation.

## Results

### Essential oil compositions

Analyzing the variance of the effects of gamma rays on essential oil composition and percentage showed that the simple effect of gamma irradiation was significant at the level of 5% on essential oil percentage. At the 5% level, the direct effect of irradiation on storage conditions and the interaction between irradiation and storage on the percentage of essential oil was significant. A significant effect was also observed based on the influence of irradiation, conditions, and duration of storage, especially on the main three compounds of *p*-cymene, thymol, and carvacrol. Additionally, it was shown that most essential oil components, particularly the three main compounds of *p*-cymene, thymol, and carvacrol, varied significantly according to irradiation, storage conditions, and duration (Table 1).

**Table 1:** Variance analysis of the effect of gamma rays × storing time × storing condition on the compounds of essential oil of *S. mutica*.

SOV	DF	EO (%)	Alpha-thujene	Alpha-pinene	*p*-Cymene	*cis*-Sabinene hydrate	Borneol	Terpinene-4-ol	Carvacrol methyl ether	Thmoquinone	Thymol	Carvacrol	E-caryophyllene	B-bisabolene	Thymo hydroquinene	Spathulenol	Catyophyllene oxide
γ (treatment)	4	*	**	**	**	**	**	**	**	**	**	**	**	**	**	**	**
Storing condition (SC)	1	ns	**	*	**	*	*	*	**	*	**	*	*	*	**	*	**
Storing time (ST)	2	ns	**	**	**	**	ns	ns	**	**	**	**	ns	ns	**	**	**
γ × SC	4	**	**	**	**	**	**	**	**	**	**	**	**	**	**	**	*
γ × SC	8	**	**	**	**	*	**	ns	**	**	**	*	**	ns	**	**	**
SC × ST	2	ns	ns	*	**	**	ns	**	ns	*	**	**	ns	**	**	*	**
γ × SC × ST	8	ns	**	**	**	**	**	*	**	**	**	**	**	*	**	*	*
cv	–	11.75	10.51	14.81	14.81	2.78	17.09	10.61	10.51	14.81	14.81	2.78	17.09	10.61	10.51	14.81	14.81

ns, * and ** indicates non-significant, significant at P ≤ 0.05 and P ≤ 0.01, respectively.

**Figure 1:**
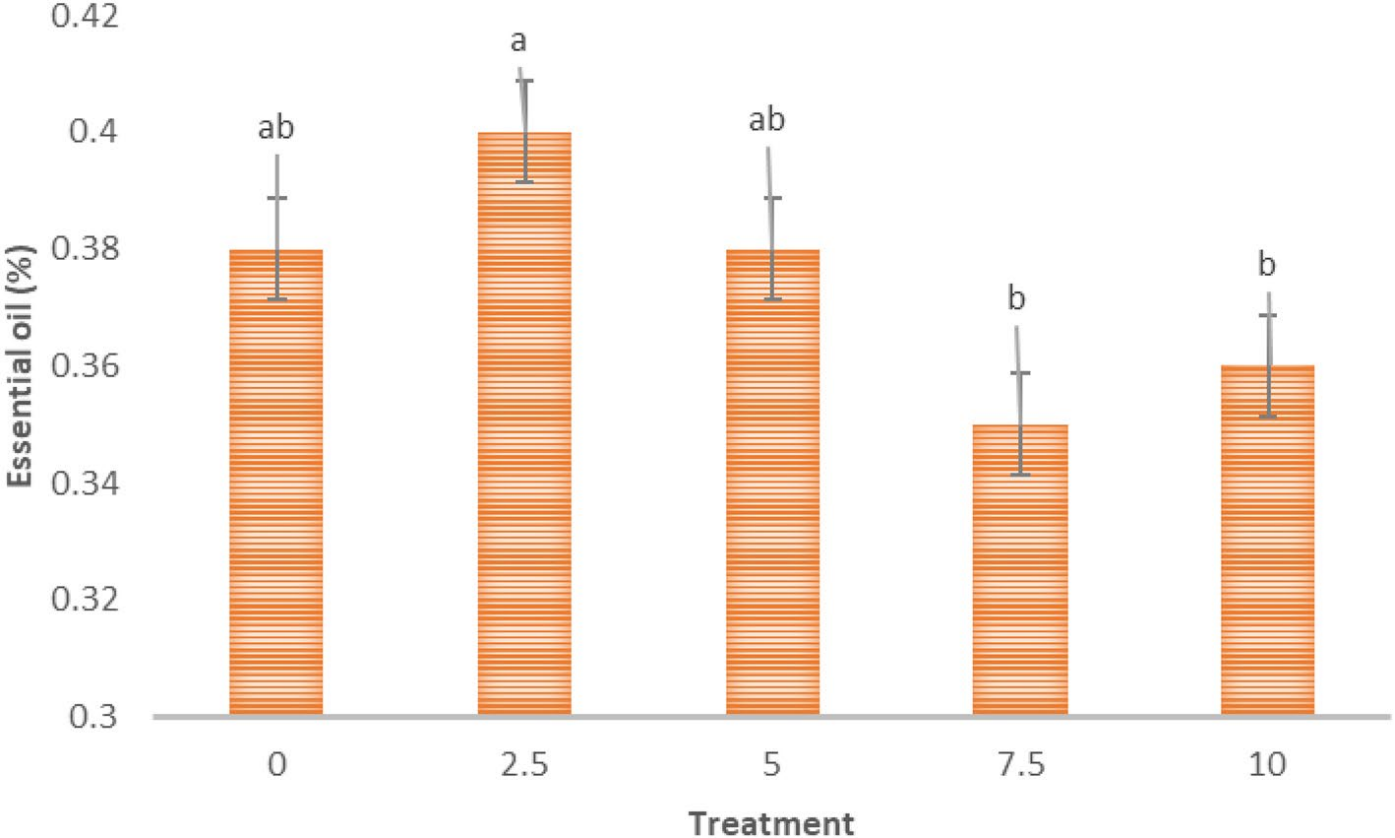
The effect of gamma ray intensity on the percentage of essential oil of *S. mutica*: means within each column followed by the same letter are not different according to the LSD test. Treatment: (0, 2.5. 7.5 and 10 kGy of gamma radiation).

As shown in Fig. 1 although there was no statistically significant difference between the 2.5 kGy treatment and the control at 5 kGy, a comparison of the means revealed that irradiation with 2.5 kGy of gamma rays produced the highest percentage of essential oil. However, as the irradiation intensity increased, the essential oil amount decreased.

**Figure 2:**
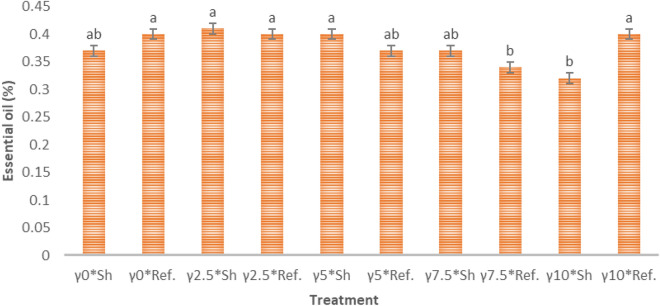
The effect of gamma radiation and storage conditions on the percentage of essential oil of *S. mutica*: means within each column followed by the same letter are not different according to the LSD test. Treatment: (0, 2.5. 7.5 and 10 kGy of γ radiation); *sh* shade, *Ref* refrigerator.

Figure 2 shows that there is no clear statistical trend.

**Figure 3:**
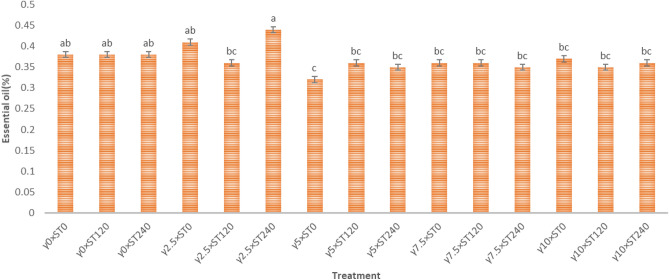
The effect of gamma ray and storage time on the percentage of essential oil of *S. mutica*: means within each column followed by the same letter are not different according to the LSD test. Treatment: (0, 2.5. 7.5 and 10 kGy of γ radiation); *ST* storing time: (0. 120, 240 h).

The treatment of 2.5 kGy in 240 h of storage had the highest percentage of essential oil, according to a comparison of the mean effects of various gamma dosages and storage periods (Fig. 3).

The amount of essential oil and the components of *S. mutica* essential oil were examined during the current study's examination of the interactions between various gamma radiation intensities, time, and storage circumstances. P-cymene, one of the important components of the essential oil and the precursor of carvacrol of *S. mutica*, had its highest amount when the plant was exposed to gamma rays at 2.5 kGy as well as its highest content in the shade and 240 h (more than 45%), so it seems that during the storage period as well as the treatment with low doses of gamma rays, the conversion of precursors to p-cymene occurs (Table 2).

**Table 2:** The effect of gamma rays × storing conditions × storing time on the components of essential oil of *S. mutica* (units of all compounds are percentage).

γ	SC (storing condition)	ST (storing time)	Terpinene-4-ol(%)	Borneol (%)	*cis*-Sabinene hydrate (%)	*p*-Cymene (%)	Alpha-terpinen (%)	Alpha-pinene (%)	Alpa-thujene (%)		
0	Shade	0	0.38bcd	0.6a	0.7a	18.15m	0.16 h	0.2 lm	0.16j		
0	Shade	120	0.14f	0.32f	0.47bcdef	14.26n	0.24gh	0.16 m	0.15j		
0	Shade	240	0.18ef	0.52abc	0.57abcd	42.71de	0.71b	0.45cdefghi	0.67def		
0	Refrigerator	0	0.41b	0.59ab	0.71a	18.46m	0.65bcd	0.43cdefghi	0.28i		
0	Refrigerator	120	0.29 cd	0.42de	0.48bcdef	45.5ab	0.57bcdef	0.46cdefg	0.66def		
0	Refrigerator	240	0.35bcd	0.5bcd	0.43def	43.44cd	0.63bcd	0.48abcde	0.84bc		
2.5	Shade	0	0.55a	0.39ef	0.62ab	43.21d	1.02a	0.58a	0.68def		
2.5	Shade	120	0.38bcd	0.5bcd	0.58abcd	41.02e	0.61bcde	0.29jkl	0.58fg		
2.5	Shade	240	0.28de	0.52abc	0.69a	47.22a	0.67bc	0.48abcde	0.89b		
2.5	Refrigerator	0	0.56a	0.51abcd	0.6a	45.02bc	0.65bcd	0.57ab	0.74 cd		
2.5	Refrigerator	120	0.33bcd	0.44cde	0.33gf	29.51 l	0.5bcdefg	0.36defghi	0.53gh		
2.5	Refrigerator	240	0.36bcd	0.45cde	0.46bcdef	45.12bc	0.58bcdef	0.59a	1.11a		
5	Shade	0	0.36bcd	0.46cde	0.35 fg	35ijk	0.33ghijk	0.34ghijk	0.2ij		
5	Shade	120	0.23bcd	0.44cde	0.52bcde	42.82de	0.53bcdef	0.53abc	0.89b		
5	Shade	240	0.39bcd	0.33f	0.21g	45.89ab	0.67bc	0.59a	1.16a		
5	Refrigerator	0	0.38bcd	0.43cde	0.38ef	34.93jik	0.33efgh	0.47abcde	0.21ij		
5	Refrigerator	120	0.29bcd	0.44cde	0.44cdef	38.22f	0.35fghijk	0.35fghijk	0.71d		
5	Refrigerator	240	0.35bcd	0.47cde	0.44cdef	42.07de	0.35fghijk	0.35fghijk	0.72d		
7.5	Shade	0	0.29 cd	0.47cde	0.48bcdef	34.36 k	0.42cdefg	0.36defghij	0.53gh		
7.5	Shade	120	0.34bcd	0.5bcd	0.72a	36.3ghij	0.45bcdefg	0.36defghij	0.42 h		
7.5	Shade	240	0.3bcd	0.47cde	0.39ef	37.67 fg	0.65bcd	0.48abcd	0.77bcd		
7.5	Refrigerator	0	0.29bcd	0.46cde	0.49bcdef	34.72jk	0.45bcdefg	0.33ijk	0.58 fg		
7.5	Refrigerator	120	0.28de	0.44cde	0.46cdef	34.16 k	0.1cdefgh	0.23klm	0.31i		
7.5	Refrigerator	240	0.35bcd	0.46cde	0.46cdef	41.05e	0.58bcdef	0.45cdef	0.85bc		
10	Shade	0	0.38bcd	0.47cde	0.39ef	35.76hijk	0.56bcdef	0.42cdefghi	0.71d		
10	Shade	120	0.37bcd	0.58ab	0.5bcdef	35.95ghijk	0.38defgh	0.33hijk	0.45 h		
10	Shade	240	0.4bc	0.48cde	0.43def	36.86fghi	0.49bcdefg	0.42cdefghi	0.58efg		
10	Refrigerator	0	0.38bcd	0.5bcd	0.41ef	35.56hijk	0.57bcdef	0.4defghi	0.68def		
10	Refrigerator	120	0.36bcd	0.45cde	0.49bcdef	34.91ijk	0.51bcdef	0.43cdefghi	0.7de		
10	Refrigerator	240	0.33bcd	0.46cde	0.36ef	37.22fgh	0.57bcdef	0.46bcdef	0.86b		

γ	CS (storing condition)	TS (storing time)	Catyophyllene oxide (%)	Spathulenol (%)	Thymo hydroquinene (%)	B-bisabolene (%)	E-caryophyllene (%)	Carvacrol (%)	Thymol (%)	Thmoquinone (%)	Carvacrol methyl ether (%)
0	Shade	0	2.6a	1.19a	2.95a	0.69cdef	1.7bcdefgh	11.9b	41.05b	7.81j	2.79cde
0	Shade	120	2.05bcde	1.07abcd	1.87e	0.52ghijk	1.6cdefgh	8.9 cd	38.37bcd	5.21 l	2.11kl
0	Shade	240	1.71efghi	0.94cdef	1.48 fg	0.66cdef	2.05abcde	7.06e	26.42ijk	9.25hi	2.51ghi
0	Refrigerator	0	2.3ab	1.15ab	3.06a	0.68cdef	1.74bcdefg	11.72b	45.87a	7.76j	3.02bc
0	Refrigerator	120	1.44hijklm	0.71ghijkl	1.17 g	5ghijk	1.19 h	6.57ef	26.29ij	8.09j	2.83cde
0	Refrigerator	240	1.33ijkl	0.74fghijkl	2.31bc	0.49hijk	1.46defgh	8.99c	32.05fgh	2.6n	2.48ghij
2.5	Shade	0	1.71fghi	0.8efghijk	1.28 g	0.68cdef	2.13abcd	6.36ef	29.19hi	2.69n	2.59efghi
2.5	Shade	120	1.84defg	0.83efghij	0.68i	0.59efghi	2abcdefg	5.9fghi	27.17ij	11.21f	2.38hijk
2.5	Shade	240	1.79defg	0.74fghijk	0.69i	0.76bcde	2.22abc	4.29kl	23.93j	8.76i	2.78cdefg
2.5	Refrigerator	0	2.003bcdef	0.89defg	1.1gh	0.87b	2.13abcd	4.82jkl	29.32hi	3.61 m	2.65defgh
2.5	Refrigerator	120	1.94cdef	0.87efghi	1.48 fg	0.77bcde	1.38defgh	17.42a	35.64cde	3.5 m	2.94 cd
2.5	Refrigerator	240	1.46hijkl	0.65jklm	1.91de	0.45ijk	1.36defgh	4.89jkl	26.24ij	9.33ghi	2.2kl
5	Shade	0	2.28abc	1.13abc	2.43b	1.1a	2.39ab	6.05fgh	39.17bc	3.33 m	3.56a
5	Shade	120	1.4ijklm	0.73ghijkl	0.67i	0.75bcde	1.59cdefgh	5.02ijkl	29.21hi	9.88j	2.76cdefg
5	Shade	240	2.11bcd	0.99bced	0.47i	0.83bc	1.61cdefgh	5.29ghi	26.27ij	6.64 k	2.72cdefg
5	Refrigerator	0	2.22bc	1.23a	2.49b	1.17a	2.09abcde	6.31ef	38.92bcd	3.36 m	3.67a
5	Refrigerator	120	1.7efghi	0.88defgh	1.86e	0.83bcd	2.06abcde	6.27efg	36.81cde	3.97 m	2.78cdefg
5	Refrigerator	240	1.47hijkl	0.73ghijkl	2.04cde	0.69cdef	2.58a	5.29fghij	34.91defg	3.38 m	2.79cdefg
7.5	Shade	0	1.29klm	0.83efghij	2.28bc	0.77bcde	1.43defgh	5.25hijk	37.13bcd	9.48gh	2.55fghi
7.5	Shade	120	1.43hijklm	0.82efghi	2.35bc	0.77bcde	1.28gh	6.46ef	36.82cde	3.4 m	2.31ijk
7.5	Shade	240	1.36ijklm	0.67ijklm	0.77hi	0.69cdef	1.76bcdefgh	5.23hijk	26.91ig	17.83b	2.54fghi
7.5	Refrigerator	0	1.2 lm	0.82efghij	2.25bcd	0.75bcde	1.42defgh	5.24hijk	37.2bcd	9.24hi	2.52fghi
7.5	Refrigerator	120	1.33jkl	0.94cdef	1.27g	0.56fghi	1.53cdefgh	4.91jkl	32.06fgh	16.48c	3.23b
7.5	Refrigerator	240	1.28klm	0.76fghijk	2.37bc	0.65defgh	1.29fgh	4.21l	31.37fgh	8.88hi	2.47ghij
10	Shade	0	1.57ghijk	0.66jklm	2.32bc	0.48hijkl	1.46defgh	6.12efgh	32.97efgh	11.78f	2.13ki
10	Shade	120	1.33jklm	0.55 lm	1.2g	4jk	1.24gh	5.96fghi	25.68ij	19.89a	2.89cde
10	Shade	240	1.68fghij	0.68hijklm	1.24g	0.52fghijk	1.44defgh	6.53ef	29.18hi	14.84d	2.11kl
10	Refrigerator	0	1.58ghijk	0.61klm	2.25bcd	0.48ijk	1.33efgh	6.21efg	33.02efgh	11.8f	2.15kl
10	Refrigerator	120	1.9m	0.54lm	1.9de	0.37k	1.27gh	8.05d	32.76fgh	12.89e	1.81 m
10	Refrigerator	240	1.4ijklm	0.51m	1.7de	0.46ijk	1.27gh	8.01d	28.98hi	13.16e	2.006kl

Means within each column followed by the same letter are not different according to the LSD test.

It can be deduced that in the event of irradiation and keeping the plant under the conditions described above, we will see a decrease in the amount of thymol. This can be related to the exit of this substance from the plant. Satureja mutica essential oil also contains thymol, responding negatively to gamma rays. At every level of this radiation, the proportion of the essential oil ingredient was minimized. However, after the plant was exposed to 2.5 kGy of gamma rays and kept in the refrigerator for 120 h, the amount of carvacrol in *S. mutica* essential oil reached its greatest value (17.42%) (Table 2).

### The impact of storage time and duration and gamma radiation exposure on the biochemical traits of S. mutica Fisch & C. A. Mey

The results of the analysis of variance of the effect of gamma rays, time, and duration of storage on the biochemical traits of *S. mutica* Fisch & C. A. Mey are given in Table 3. As can be seen, each of these has a considerable impact on biochemical traits such as phenol content, antioxidant capacity, and carbohydrate. Furthermore, it affects the interaction between gamma rays × storage time × duration. These findings showed that the phenol content rose over time, reaching its peak (16.76 µg/ml) after 240 h of storage in the shade with 2.5 kGy of gamma radiation. The amount of total phenol dropped as the gamma radiation dosage increased. As a result of a high radiation dosage, lower phenol content was extracted so that the least of it was observed at 10 kGy gamma radiation (10.33 µg/ml) in refrigerator storage after 240 h (Table 3).

**Table 3:** The effect of gamma rays × storing conditions × storing time on the biochemical traits of *S. mutica*.

γ	CS (storing condition)	TS (storing time)	Phenol (µl/ml extracted)	Antioxidant (%)	Carbohydrate (µl/ml extracted
0	Shade	0	11.43fgh	35.45q	8.66a
0	Shade	120	11.8efg	32.38q	0.7.65b
0	Shade	240	12.36defg	38.01p	6.52cd
0	Refrigerator	0	12.5defg	50.74n	6.5cd
0	Refrigerator	120	12.28defg	52.48lm	0.48bcdef
0	Refrigerator	240	12.66de	53.46l	7.43b
2.5	Shade	0	12.74de	61g	6.6c
2.5	Shade	120	16.69ab	55.62k	0.58abcd
2.5	Shade	240	16.76a	56.43jk	5.6def
2.5	Refrigerator	0	15.64abc	57.88ij	5.66de
2.5	Refrigerator	120	14.55c	59.73gh	5.53efgh
2.5	Refrigerator	240	16.63ab	61.02g	0.46bcdef
5	Shade	0	15.53bc	50.89n	5.46efghi
5	Shade	120	15.56bc	51.36mn	5.54efghi
5	Shade	240	16.61ab	65.6e	5.43efghij
5	Refrigerator	0	16.57ab	63.6f	4.43k
5	Refrigerator	120	16.69ab	58.78hi	4.37k
5	Refrigerator	240	16.52ab	51.38 mn	4.61ghijk
7.5	Shade	0	13.43d	43.78o	4.55ijk
7.5	Shade	120	14.57c	44.43o	4.69fghijk
7.5	Shade	240	12.53def	66.95e	4.56ijk
7.5	Refrigerator	0	12.7de	69.66d	4.37k
7.5	Refrigerator	120	12.34defg	74c	3.96kl
7.5	Refrigerator	240	12.64de	85a	3.71kl
10	Shade	0	11.43 fg	51 mn	5.56efg
10	Shade	120	11.78efg	55.53 k	4.6hijk
10	Shade	240	11.35gh	57.63ij	4.5jk
10	Refrigerator	0	10.5h	63.46f	4.44k
10	Refrigerator	120	10.49h	69.9d	3.8kl
10	Refrigerator	240	10.33h	77.5b	3.4l

Means within each column followed by the same letter are not different according to the LSD test.

Under conditions of refrigerator storage, 240 h of storage period, and 7.5 kGy of gamma radiation, the highest concentration of antioxidant capacity was discovered. After 240 h, 10 kGy treatment showed a significant difference in antioxidant capacity (77.5%) from the control, whereas 5 kGy treatments showed a marked decline in antioxidant capacity (51.38%) (Table 3).

Table 3 shows that the radiation-free condition had the highest carbohydrate content. Additionally, the findings indicated that carbohydrates decreased during storage time. Nonirradiated samples exhibited a higher total carbohydrate content (average values considering all irradiated materials (8.66 µg/ml extracted) than the corresponding irradiated samples). In particular, depending on the increased radiation dose, carbohydrate levels decreased significantly. Irradiated samples exhibited lower total carbohydrate content (average values considering 10 kGy irradiated materials in the refrigerator for 240 h = 3.4, 3.7, and 4.61 µg/ml extracted, respectively), while lower radiant (5 and 10 kGy) resulted in a significant increase in carbohydrate levels.

## Discussion

Research results show that nuclear technologies significantly affect morphology and phytochemistry to boost agricultural production and improve quality. Several researchers have compared the active components of irradiated medicinal plants with control samples, but only a small number of those studies have used high intensities to detect changes in the principal constituents of the treated plants ^23^. Gamma radiation is often called a cold process in which the processed material's temperature does not significantly increase. This method is, therefore, suitable for heat-sensitive items like medicinal plants.

Based on this study, the essential oil (EO) percentage obtained from the aerial parts of *S. mutica* Fisch & C. A. Mey with 2.5 kGy gamma radiation was higher. In addition, it was affected by 2.5 kGy gamma ray doses and storage duration after 240 h.

The present study describes the essential oil profile and biochemical changes in the main compounds of p-cymene, thymol, and carvacrol that emphasize the appropriate treatments and subjects’ selection; it also shows that drying before hydrodistillation resulted in losses or increases of essential oil, so the issue has been further investigated. The subject and treatments were appropriately chosen when there were noticeable differences between treatments in the percentage of essential oil and the main compounds of p-cymene, thymol, and carvacrol ^15^.

In agree with our results, the content of bioactive components increased in Artemisia ^24^, white mushrooms ^25^, and *Carum carvi* L. ^26^ when exposed to gamma rays. Although, high intensities have a decreased yield in plants like barley ^27^, but low doses have an increased yield. As a result, to increase plant performance, one should act after knowing the impact of gamma radiation on the intended product and its optimal intensity level. According to Haddad et al. ^28^, low levels of gamma rays positively affect the percentage and composition of essential oils from medicinal plants like thyme, lavender, and eucalyptus. This research had similar results to this research. These outcomes are consistent with those reported by *Tanacetum annuum* L. ^29^, *Thymus vulgaris* L. ^30^; *Syzygium aromaticum* and *Cinnamomum zeylanicum* ^31^.

According to our finding**, **16 essential oils were extracted from *S. mutica* Fisch and C. A. Mey after gamma radiation. Carvacrol and P-cymene make up the majority of the oil. The essential oil of *S. mutica* Fisch & C. A. Mey gathered from north and northwest Iran has 48 and 46 components, respectively ^32^. Thymol was the highest component in the two groups identified, followed by p-cymene, terpinene, and carvacrol. Previous research produced identical results ^33^.

In the current study, storage conditions affected some chemical compounds' ratios of p-cymene, carvacrol, and thymol. Moreover, the highest carvacrol content was produced from 2.5 kGy of radiation at shade. Additionally, savory showed a significant increase in thymol in refrigerator condition. The fact that the amount of thymol was higher in the refrigerator than in the shade suggests that by adjusting plant storage conditions**,** one may keep a compound constant, reduce it, or increase it. The amount of p-cymene grew, and the amount of carvacrol decreased as plant storage time increased, which can be a crucial management point in creating the potent chemical. In other words, the quality of the essential oil (carvacrol) was diminished by using gamma rays for a longer time. The main essential oil constituents have a negative correlation, meaning their levels decrease as one increases. Therefore, duration and storage requirements, as well as corrective actions, will play a crucial role. The lack, or small effect, of irradiation on thyme aroma compounds agrees with Pereira et al. ^34^.

According to our findings, gamma radiation significantly affected phenolic compounds and antioxidant activity. In addition, other compounds react and change besides essential oil as a volatile substance that evaporates through transpiration and humidity. Phenol is one of the antioxidant chemicals, and in this experiment, the stress condition was thermal stress. Phenol production increases under stress conditions, but as stress intensity increases, another pathway is activated, and phenol production decreases. According to Abbaszadeh et al. ^35^, the proportion of essential oils and some physiological compounds rises under mild stress. As stress severity increases, new pathways become available, and the number of new compounds in the plant rises. A drop in phenol concentration at high radiation illustrates the same problem. Gamma rays have been shown to disrupt hormones, gas production, water absorption, and leaf enzyme activity. These actions modify plant metabolism, photosynthesis, cell structure, and phenolic chemical accumulation. In contrast, chloroplasts are significantly more sensitive to gamma rays than other cellular organelles, and the rise in pigment is very noticeable in samples exposed to radiation^17^. Due to the high concentration of phenolic and flavonoid components in the low-rate irradiation treatment, the highest antioxidant activity was seen.

In this regard, Kosalec et al. ^36^ found no appreciable differences in the amount of phenols and essential components of rosemary, artichoke spring grass, and basil by using the various gamma-ray intensities. Ebadi ^23^ stated that radiation had no discernible impact on the proportion of essential oils and their constituents, such as limonene and citral. In addition, the amount of antioxidants and total phenol was higher in the control samples. Gamma radiation at doses of 10 kGy on thyme ^30^, 2 kGy on Stevia ^37^, and 10 kGy on *Thymus algeriensis* ^38^ had a significant impact on essential oil alterations. Our findings agree with the study reporting that radiation less than 10 kGy does not alter carbohydrates ^39^^.^

As a general statemen, essential oils with high concentrations of p-cymene and carvacrol are generally created using gamma-ray treatment with an intensity of 2.5 kGy in the relevant industries. Using a plant that has just been harvested may be a better choice if the thymol concentration is high. Changes in essential oil compositions (carvacrol and thymol para-cymene) were detected during storage, confirming that storage in medicinal plants is critical and that future research could be addressed. The decrease in thymol in the shade compared to the refrigerator indicates that preservation, increasing or decreasing EOs might be achieved by choosing plant storage conditions. Despite widespread publicity regarding the effectiveness of gamma rays in various plants, prospective studies may provide the most accurate information regarding the cost of transmission and the disadvantages of gamma rays on a large scale in the savory.

## Data Availability

The datasets used or analyzed during the current study are available from the corresponding author upon reasonable request**.**
